# Unveiling role of oncogenic signalling pathways in complicating breast cancer

**DOI:** 10.37796/2211-8039.1640

**Published:** 2025-03-01

**Authors:** Acharya Balkrishna, Sagar Kumar, Rohan Malik, Kuldeep Singh Mehra, Hariom Chaturvedi, Okeshwar, Rashmi Mittal

**Affiliations:** a Patanjali Herbal Research Department, Patanjali Research Institute, Haridwar, India; b Department of Yog Science, University of Patanjali, Haridwar, India; c Department of Sanskrit, University of Patanjali, Haridwar, India

**Keywords:** JAK/STAT, MAPK, PI3K/AkT/mTOR, NF-κB, Subtypes, US-FDA

## Abstract

Heterogeneous nature of breast cancer has significantly affected the overall survival, disease free survival and progression free survival amongst the diseased individuals. Metastasis of cancerous cells to distant sites including bone, lungs, liver, lymph node and others have further exhilarated the adverse effects. However, ER, PR and HER-2 are responsible for normal physiological development of women but in altered conditions they may act as initiator or progressor and so far 5 subtypes of disease have been identified. Alteration of pro-survival, pro-proliferative and anti-apoptotic pathways including JAK/STAT, MAPK, PI3K/AkT/mTOR, NF-κB, BCL2 and several others have induced oncogenic events including epithelial-mesenchymal transition, intra-vasation, extra-vasation and many more. Although several US-FDA approved drugs are available in market to target above mentioned signalling pathways but issues of resistance, side effects have restricted their efficacy. The present review article aims to highlight diverse molecular subtypes and the signalling pathways involved in complicating the disease along with the US-FDA approved drugs to target them. Potential herbal medicine to target the disease have also been emphasized that can be used either as mono-therapeutic approach or in combination with conventional therapeutic regimens to target breast cancer.

Breast cancer (BC) is one of the most common malignancies amongst women worldwide and it cannot be considered as single disease and it is characterized by distinct pathological and molecular subtypes [1]. The abnormal proliferation of the breast's lobular and ductal epithelium, which turns malignant and forms a tumor, is referred as breast cancer, a multifactorial and incredibly heterogeneous disease. Metastasis and diseases recurrence issue has significantly led to the poor overall survival, disease free survival and distant metastasis free survival rate. Fortunately, early detection and effective systemic therapies have led to a decrease in breast cancer mortality in North America and the European Union in recent years. On the basis of gene expression profiling breast cancer has been classified into 5 molecular subtypes including luminal A, luminal B, HER-2 enriched, basal-like and claudin-low. The expression of several biomarkers including the human epidermal growth factor receptor 2 (HER-2) status, progesterone receptor (PR), proliferation marker Ki-67, and estrogen receptor (ER), can also be used to classify overlapping subtypes [2,3].

However, up to ten distinct molecular subtypes have been discovered through molecular subtyping and expression analyses so far. Moreover, intratumoral heterogeneity enhances the potency of cancer cells to change their behaviour and alter their gene expression profile in response to the surrounding environment. Continuous alteration in gene expression profiling of cancer cells have adversely affected prognosis and treatment strategies. Indeed, once a cancer has initiated locally, may spread to other organs like the brain, liver, lungs, or bones, thereby making it invasive this has further leads to increase in mortality rate worldwide [4,5].

As a result, there are distinct forms of breast cancers, each with a unique location, histological type, molecular signature, and, most importantly, reliance on hormones for growth and development. These variables combine to cause a wide range of aggressiveness and prognosis in breast cancers, necessitating different approaches for therapeutic management. So far several drugs have been approved to combat BC (Fig. 1) [6–9].

Oxidative stress also played a significant role in initiating events of carcinogenesis especially in tumor microenvironment induced by chemo-therapeutics. Oxidative stress can induce cancer stem cell growth, epithelial-mesenchymal transition, increases glutathione and nuclear factor erythroid 2 related factor 2 [10,11].

Extensive research is being conducted to identify additional biomarkers, which may include single proteins, panels of proteins/mRNAs, or aberrantly expressed pathways within the cancer cell that may facilitate diagnosis, prognosis, and treatment of primary breast cancer and metastatic events [12]. The present review article aims to highlight various subtypes of breast cancer based on their hormonal status and oncogenic signalling pathways involved in complicating the disease along with a panorama of US-FDA clinically approved drugs and unleashed the potency of herbal medicine to combat the disease.

## 1. Classification of breast cancer

World Health Organization (WHO) guidelines are updated periodically to enhance patient care, grading of cancer and selecting the best course of treatment to defeat the illness. One way to determine whether hormone therapy or targeted therapies may exhibit promising results or not, is to look at the presence or absence of hormone receptors that may serve as potentially targeted site for the treatment. On the basis of molecular expression profile, 4 major subtypes of breast cancer were determined named as luminal A, luminal B, HER-2 positive and basal like triple negative tumor. Another subgroup that resembles the luminal A group but has a worse prognosis has also been described as normal-like on virtue of their similar genetic profile. Claudin-low have also been considered as 5^th^ subtype of breast cancer [13–17].

ER, PR, and HER-2 are immune-histochemical markers that are commonly used in clinical practice to classify diverse tumor sub-types especially on the basis of hormonal positive and hormonal negative BCs. In fact, transcriptomic data that identifies the various forms of breast cancer can be replaced by these tumor identification biomarkers for prognostic and therapeutic purposes. The least aggressive cancers are those that express ERα, which is generally a characteristic of luminal cancer type. Whereas luminal type B expresses both HER2, and PR [18].

Because of a higher expression of genes related to cell proliferation and the cell cycle, such as Ki-67 and Aurora kinase A, luminal B cancers are therefore more aggressive than luminal A (low proliferative) cancers. Moreover, the expression of genes linked to luminal growth, like PR and FOXA1, is significantly reduced in luminal B type. Luminal B tumors are linked to a higher rate of p53 mutations (29 % vs. 12 %), in contrast to luminal A types. The expression of PR and ERα receptors is absent in HER-2 positive cancers. This favours the progression of metastasis and thus makes this type of breast cancer more aggressive than luminal cancers. Basal-like tumors that do not express the major biomarkers (ERα, PR and HER2) were therefore defined as triple negative in relation to the other subgroups. Elevated expression of keratin 5, keratin 17, intergrin-B4, laminin, MYC, CDK6, and CCNE1 are among the gene expression characteristics of this breast cancer subtype [19,20].

A high TP53 mutation rate and altered DNA repair, including the deletion of BRCA2, PTEN, and MDM2, are additional characteristics of triple-negative breast cancer. These tumors have a poor prognosis because they are more aggressive and heterogeneous, exhibits a high histological grade, proliferation index, and a high local and distant recurrence rate. In this section of the article we will briefly discuss the different subtypes of breast cancer based on ER, PR and HER2 expression [21–23].

### 1.1. Luminal A subtype of breast cancer

The presence of ER/PR, absence of HER2, and the low expression profile of the cell proliferation marker Ki-67 (less than 20 %) are the basic characteristics of luminal A subtype of BC. Clinically speaking, they are low grade, slow growing, and exhibited the best prognosis accompanied with higher survival rate and lower relapse rate. Chemotherapy has a more restrictive effect on these carcinomas, while hormone therapy (tamoxifen or aromatase inhibitors) showcased a high response rate. Therefore, it is highly needed to determine which patients group would get benefit from adjuvant chemotherapy treatment based on the risk of relapse and survival rate. For this, the use of genetic platforms is advised by the European Society for Medical Oncology (ESMO) and the National Comprehensive Cancer Network from USA (NCCN). Disease relapse are less common in the central nervous system (CNS) and visceral regions, and more profoundly is observed at the bone level. In the event of a disease relapse, the chances of survival also get affected that ultimately impacted the mortality rate caused due to the disease. But in comparison to other BC subtypes, luminal A subtypes are least aggressive [24,25].

### 1.2. Luminal B subtype of breast cancer

When compared to Luminal A tumors, Luminal B exhibited worst prognosis and a higher grade. They have a high expression of Ki-67 (more than 20 %) and are ER positive, and PR negative. Typically, they have an intermediate to high histologic grade. In addition to chemotherapy, hormonal therapy may also display beneficial outcomes. Because of their higher Ki-67 index, they grow more quickly than luminal A and exerts worst prognosis. Genes related to proliferation and cell cycle were observed to be elevated, whereas the oestrogen receptor expression is moderately low. This signifies the category of luminal tumors that have the most unfavourable outlook. In comparison to the preceding group, they achieve greater benefit from hormone therapy and chemotherapy. The rate of visceral recurrence is relatively higher, and the survival rate from diagnosis to relapse is shorter, despite the frequent recurrence at the bones.

### 1.3. HER2 subtype of breast cancer

HER-2 positive BCs has been linked to an aggressive phenotype and an unfavourable outcome of the disease. But the prognosis of these patients and the course of the disease were drastically altered with the advent of HER2 targeted therapies. Long-term follow-up data showed that even with the improvement, 15–23 % of patients in the early stage still experience disease recurrence. Out of all BC patients, 15 % overexpressed HER2, and roughly 50 % of these are also hormone receptor positive (HR+), meaning that HR+/HER2 have significant biological and clinical differences. Approximately thirty percent of HR+/HER2+ BCs are HER2161 Enriched (HER2-E), indicating high molecular heterogeneity. High HER2/EGFR pathway activation, increased proliferation, and an immune-activated stroma with elevated tumor infiltrating lymphocytes are the characteristics of this subtype. Compared to the Luminal A and B subtypes, it expresses fewer genes involved in complicating the disease, which means it may benefit greatly from anti-HER2 therapies but poorly from endocrine therapy (ET) [26].

### 1.4. Hormonal receptor negative subtype of breast cancer

Triple Negative Breast Cancer (TNBC) lacks the expression of ER, PR and HER2. On the basis of gene expression profiling analysis, TNBCs are often referred as basal like BCs. Approximately 56 % overlap was identified between BLBCs and TNBC subtype whereas between non-TNBC and BLBCs, the ratio constitutes about 11.5 % [27,28].

Due to its high level of invasiveness, 46 % of TNBC patients will experience distant metastasis. Only 13.3 months is the median survival time after metastasis, and up to 25 % of cases recur after surgery. The brain and visceral organs are frequently affected by the metastasis. Most distant metastases happen in the third year following diagnosis. For non-TNBC patients, the average time for disease relapse is 35–67 months, whereas for TNBC patients, it is only 19–40 months. Up to 75 % of TNBC patients passed away within three months of disease recurrence [29]. TNBC is non-responsive to molecular targeted therapy or endocrine therapy because of its unique molecular phenotype. Chemotherapy is therefore the principal systemic treatment. However, traditional post-operative adjuvant chemoradiotherapy is not very effective. Tumor recurrence would eventually result from the remaining metastatic lesions. In certain nations, bevacizumab has been used to treat TNBC in conjunction with chemotherapy medications. However, patient survival duration did not significantly improve even after the chemotherapy and surgery. Consequently, it is imperative to create new treatment plans and objectives. Henceforth, treatment of TNBC is a cumbersome task [30,31].

## 2. Signalling pathways involved in complicating breast cancer

Cancer develops and spreads through a fundamental process referred as cell transduction. Variations in the signaling pathways of cells encourages the growth, survival, motility, and multiplication of tumor cells and alters the regulation of immune response. One of the primary factors contributing to cancer cells' resistance to anti-tumor treatments is the activation of signalling pathways including NF-κB, PI3K/AkT/mTOR, JAK/STAT, MAPK, Wnt/β-Catenin and Notch signaling Pathways. Activation of these pathways further affected the prognosis and diagnosis in addition to serving as a possible therapeutic target. In this section of the article, we will briefly discuss the above mentioned signalling pathways and their role in complicating the disease [32,33].

### 2.1. Role of NF-κB signalling pathway in complicating breast cancer

Apart from involvement of hormones, transcription factors also played a significant role in complicating breast cancer including nuclear factor kappa B (NF-κB). This transcription factor is localised primarily in nucleus and is involved in development and progression of diseases. Moreover, inflammatory breast cancer (IBC), an ER-negative and HER2-positive subtype of BC that is particularly aggressive, is specifically linked to NF-κB activation. When NF-κB signalling is over-expressed, either on its own or in combination with other signalling pathways, it can induce angiogenic neo-vascularization, the epithelial-mesenchymal transition (EMT), make cancer cells more “stem-like,” resistant to chemotherapy, radiation, and endocrine disruption. A reduced overall survival rate, advanced forms of the disease, and early relapse are all linked to these invasive phenotypes. Furthermore, NF-κB is activated by the widely used cancer chemotherapy and radiation treatments, which results in the development of invasive BCs that are resistant to these treatments as well as endocrine therapy. When NFκB is inhibited, cancer cells become more susceptible to the apoptotic effects of radiation and chemotherapy [34–41]. In HER2 tumorigenic mouse model of breast cancer, it was observed that selective inhibition of NF-κB substantially regulated the initiation & proliferation of cancerous cells, inflammation, recruitment of TAMs and colony formation in mammary gland. Similar observations were also experienced in c-Rel transgenic mouse model. Elevated expression of NF-κB was observed upon the development of breast cancer in c-Rel mouse. This further enhanced the expression of associated targeted genes including Cyclin D1, Bcl-xl and c-Myc. From these evidences, it can be clearly predicted that NF-κB in directly involved in enhancing the complications of breast cancer [42,43].

However, several pre-clinical and clinical investigations were carried out to search for potential therapeutic candidates to target NF-κB. Along with conventional therapeutic regimens, curcumin obtained from turmeric is well known to inhibit metastasis in BC by targeting NF-κB [44,45].

### 2.2. Role of PI3K/AkT/mTOR signalling pathway in complicating breast cancer

The complex intracellular pathway known as PI3K/Akt/mTOR promotes cell growth and tumor proliferation and is a major responsible factor in endocrine resistance in breast cancer. A number of drugs that target this pathway are being tested in pre-clinical and clinical trials. PI3K/Akt/mTOR was observed to be crucial for the growth and multiplication of tumor cells. It reacts with the availability of nutrients, hormones, and growth factors. Endocrinological resistance has been linked to the PI3K/Akt/mTOR pathway. Pre-clinical research has demonstrated that Akt can activate the estrogen receptor (ER) pathway regardless of the availability of estrogen and that mTOR inhibitors in conjunction with endocrine therapy can overcome resistance to endocrine therapy in BC that is HR+. In HER2-overexpressing breast cancer, the PI3K/Akt/mTOR pathway has also been connected to trastuzumab resistance.

*In vivo* studies indicated that Akt1-E17K mutation may induce mammary hyperplasia that can eventually lead to lung epithelium disorder in mouse models thereby indicating their oncogenic role in initiation and development of tumor [46].

In another study, impact of PI3K mutations in inducing events of oncogenesis was determined in genetically engineered mouse models. It was observed that oncogenic mutation in PI3K is directly associated with either activating subsequent mutations in PIK3CA or Akt1 or is linked with decreased expression of PTEN thereby further complicating breast cancer [47].

Pre-clinical research suggested that in resistant cells, pathway inhibitors can function in conjunction with trastuzumab as well. Numerous PI3K/Akt/mTOR pathway inhibitors are currently in pre-clinical development or are currently undergoing clinical trials. Promising evidence suggested that rapalogs or PI3K/Akt inhibitors are effective to treat breast cancer [48–50]. In mouse models, buparlisib exerted anti-tumor activity with enhanced oral bioavailability under *in vivo* conditions. Flavonoids including curcumin, quercetin, formononetin, saponins and non-flavonoids including resveratrol and anthricins are well known to target PI3K/Akt/mTOR pathway in BC. Their formulations need to be modified to further enhance their bioavailability [51–56].

### 2.3. Role of MAPK signalling pathway in complicating breast cancer

Aggressive BCs have been found to exhibit MAPK signalling activity in multiple studies. One of the pathways, which is mediated by HER-2 and the epidermal growth factor receptor (EGFR), activates MAPK signalling in BCs. Thus, MAPK interacting kinase signalling is stimulated by activated ERK, and MNK in turn activates XIAP, a downstream protein. An apoptotic inhibitor protein called XIAP is upregulated in aggressive and inflammatory BC and connects MAPK signalling to NFκB signalling activity. Conversely, IAP increases the expression of the EMT factor Snai2, which is linked to enhanced stem cell characteristics. IAP protein inhibition reduces MAPK activity by downregulating the expression of Snai2 and EGFR. Hypoxia-inducible factor-1 (HIF1)-dependent signalling pathways are involved in one of the other MAPK activating signalling pathways in BC resistance to chemotherapy. The expression of dual specificity phosphatase (DUSP9) and DUSP16 is regulated by chemotherapy in a manner that is dependent on HIF1. In turn, a decrease in DUSP16 expression activates the p38 signalling pathway while an increase in DUSP9 expression inhibits ERK. Through decreased phosphorylation of FoxO3, ERK inhibition increases the expression of Nanog. p38δ MAPK plays a pivotal role in the development of cancer cells, their metastasis, and the control of BCSC phenotypes and EMT. EMT mediated by p38δ MAPK is enhanced through the suppression of miR200b, which is downstream of p38γ MAPK. Through the induction of GATA3 ubiquitination and its subsequent degradation, p38γ MAPK inhibited miR200b [57].

In BC, ligand independent actions of phospho-SRs are generally promoted by MAPK signalling pathways. This eventually leads to activation of cell proliferation by inducing transcription of PR targeted genes MYC and CCND1 in mouse mammary tumor models. In immuno-deficient mouse model, it was observed that active MAPK Kinase 3 with reduced copy number increased the expression of CDK inhibitor p^21^ and p^27^ thereby arresting the cell cycle at G1 phase. From these observation, it can be concluded that MAPK Kinase 3 suppresses the tumor growth in breast cancer when expressed with reduced copy number [58]. Naringenin obtained from tomatoes and citrus fruits is also under evaluation to target MAPK against breast cancer [59].

### 2.4. Role of Wnt/β-catenin and notch signalling pathway in complicating breast cancer

Apart from NF-κB, MAPK, PI3K/Akt/mTOR and several other signalling pathways implicated in the maintenance of breast cancer stem cells included Wnt and Notch. Hypoxia mediates HIF 2α overexpression, which can activate Wnt and Notch signalling pathways. Over-expression of HIF-2α leads to drug resistance in BCSCs, overexpression of BCSC markers, and phenotypic conversion of stem cells. Poor patient survival rate is correlated with over-expression of glycogen synthase kinase 3 beta (GSK3β), one of several factors implicated in Wnt signalling. Programmed death 1 (PD-1), which is overexpressed in BCSCs, mediates the contribution of Wnt signalling in stem-ness phenotypes of CSCs. Furthermore, PD-1 is up and down-regulated by Wnt activators and inhibitors respectively. Certain characteristics of stem cells are linked to high expression of PD-1. According to recent reports, BCSCs' Wnt/β-catenin was regulated in part by kinesin family member 11 (KIF11), a motor protein required for mitosis. BCSCs' stem-ness phenotypes are enhanced by activating Wnt/β-catenin. Nectin-4 is the other protein that controls Wnt/β-catenin in BCSCs. A well-known BCSC marker, nectin4 is a junction protein that is connected to BC and BCSC metastasis. Nectin-4 overexpression enhances the Wnt/β-catenin signalling pathway, which is connected to BCSC proliferation, metastasis, and EMT.

In transgenic mouse model, impact of Wnt signalling in initiation and progression of breast cancer was evaluated. By inserting MMTV-LTR upstream of Wnt1 gene, transgenic mouse mammary model was established. In 6 months, ductal hyperplasia was observed, that eventually leads to formation of breast cancer by activating β-catenin signalling pathway [60,61]. In mouse mammary tumor virus model, elevated expression of Notch4 was observed to be predominantly involved in complicating breast cancer. Notch were predominantly implicated in epithelial-mesenchymal transition and in inducing hypoxia [62]. Henceforth, it is highly essential to develop Wnt/β-Catenin and Notch inhibitors to combat the disease. However epigallocatechin gallate is currently under pre-clinical studies to evaluate its efficacy to target above mentioned signalling pathways [63].

## 3. US-FDA approved drugs for treatment of breast cancer

The Department of Health and Human Services oversees the United States Food and Drug Administration (US-FDA), a federal agency that controls the approval process for drugs to be used in humans and animals as well as a variety of other products. 39 medications, out of the 207 total that the FDA has approved for use in oncology, are expressly approved for use as a single or adjuvant treatment for breast cancer. More drugs for breast cancer indications have been approved by the FDA in the past 70 years than for any other type of cancer. Most of these medications were initially authorized for the treatment of metastatic breast cancer. Subsequently, about 31 % of medications were granted additional adjuvant status. So far, several inhibitors have been evaluated both pre-clinically and clinically to target above mentioned signalling pathways (Table 1) [64–80].

## 4. Role of herbal medicine in treating breast cancer

Natural plant products, including flavonoids, alkaloids, terpenoids, and coumarins, are recognized for their potent immune-modulatory qualities that are required to suppress or combat cancer cells. These constituents include glabridin, curcumin, arctigenin, and ajoene, as well as quinic acid, β-carotene, epigallocatechin-3-gallate, and ginsan. Hormonal issues are often the cause of these cancer outcomes, and bioactive substances such as phytoestrogens (non-steroidal phenolic compounds with structural similarities comparable with steroids like oestrogen) and iso-flavonoids can act as endocrine disruptors for these disorders. It has been reported that these plant flavonoids have chemo-preventive, estrogenic, and/or anti-estrogenic properties. They have the capacity to initiate oxidative stress and to induce cancer through oestrogen receptor signalling. They can also inhibit oestrogen receptor dependent (cell growth and proliferations) and independent (generation of free radicals and genotoxic agents) associations. Research has demonstrated the effectiveness of natural products, like herbs, in the preparation of anticancerous medications. Ginseng, Garlic (*Allium sativum*), Black cohosh (*Actaea racemose*), Turmeric (*Curcuma longa*), Green tea (*Camellia sinenis*), Flaxseed (*Linum usitatissimum*), and Black cumin (*Nigella sativa*) are herbs with chemo-preventative and chemo-therapeutic qualities. In above mentioned sections, we already discussed the potency of phytochemicals to target signalling pathways including NF-κB, MAPK, PI3K/Akt/mTOR, Wnt/β-Catenin and Notch Signalling. These herbs also possess several biochemical and pharmacokinetic characteristics. Popular herbs that are frequently used as adjuvants in breast cancer therapy in traditional medicine have been studied, and their mechanisms of action have also been explored. More research needs to be carried out in this domain to further unleash the potency of herbal medicine to target different breast cancer subtypes and the signalling pathways involved in complicating the disease [81–83].

## 5. Conclusion

Despite of several pre-clinical and clinical investigations, mortality rate caused due to the disease is relatively higher. Understanding of signalling pathways have remained to be a crucial subject for development of effective therapeutic regimens. Candidates concomitantly targeting them may emerge as substantial therapeutic agents. There exists an urgent need to work on combinational therapeutic approach comprises of synthetic analogs and phytochemicals to reduce the events of resistance, re-occurrence and side effects and to further improve the overall survival and disease free survival rate amongst the diseased individuals.

## Figures and Tables

**Fig. 1:**
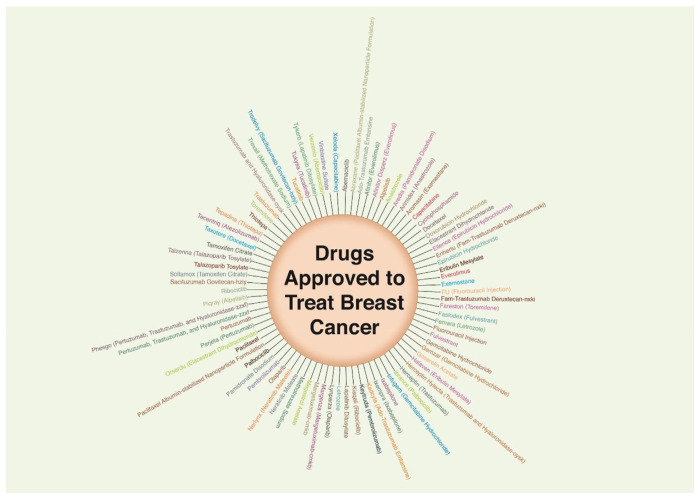
Figure representing several drugs clinically approved to treat breast cancer.

**Table 1:** List of drugs along with targeted pathway, clinical trial number and their status 319 currently under investigation against breast cancer.

Drug	Targeted Pathway	Clinical Trial Number	Status
Olaparib	PARP	NCT02000622	Phase III
Olaparib + Carboplatin	PARP	NCT01445418	Phase I/II
Olaparib/Eribulin	PARP	UMIN00009498	Phase I/II
Olaparib + Durvalumab	PARP	NCT02734004	Phase I/II
Palbociclib	CDK4/6	NCT02513394	Phase III
Palbociclib + Fulvestrant	CDK4/6	NCT01942135	Phase III Trial
Ribociclib + Letrozole	CDK4/6	NCT01958021	Phase III
Abemaciclib	CDK4/6	NCT01394016	Phase I
Abemaciclib + Fulvestrant	CDK4/6	NCT02107703	Phase III
MK-2206 + Trastuzumab	AKT	NCT00963547	Phase I
Capivasertib	AKT	NCT01226316	Phase I
Capivasertib + Fulvestrant	AKT	NCT01992952	Phase II
Capivasertib + Paclitaxel	AKT	NCT01625286	Phase I/II
Ipatasertib + Paclitaxel	AKT	NCT02162719	Phase II
Bevacizumab + Paclitaxel	VEGF	NCT00028990	Phase III
Bevacizumab + Trastuzumab	VEGF	NCT00391092	Phase III
Ramucirumab	VEGFR	NCT01234402	Phase II
Cediranib + Fulvestrant + Docetaxel	VEGFR	NCT00454805	Phase II
Sorafenib + Gemcitabine/Capecitabin	VEGF	NCT00493636	Phase II
Dovitinib + Fulvestrant	FGF/FGFR	NCT01528345	Phase II
Lucitanib	FGF/FGFR	NCT02202746	Phase II
Lenvatinib	FGF/FGFR	NCT03168074	Phase II
Infgratinib + Alpelisib	FGF/FGFR	NCT01928459	Phase I
AZD4547 + Letrozole/Anastrozole	FGF/FGFR	NCT01791985	Phase II
GP369, FPA144, MFGR1877S	FGF/FGFR	NCT00687505	Phase I
Cemiplimab	PD-1/PD-L1	NCT04243616	Phase II
Cemiplimab + LAG-3 Inhibitor + Paclitaxel	PD-1/PD-L1	NCT01042379	Phase II
Pembrolizumab	PD-1/PD-L1	NCT01848834	Phase II
Pembrolizumab + Standard Chemotherapy	PD-1/PD-L1	NCT02819518	Phase III
Eribulin + Pembrolizumab	PD-1/PD-L1	NCT02513472	Phase Ib/II
Cisplatin + Doxorubicin	PD-1/PD-L1	NCT02499367	Phase II
Cryoablation/Ipilimumab	CTLA-4	NCT01502592	Phase I
AE37 + GM-CSF Immunotherapy	HER2	NCT00524277	Phase II

## Abbreviations

BC: Breast Cancer
HER-2: Human Epidermal Growth Factor Receptor 2
PR: Progesterone Receptor
ER: Estrogen Receptor
WHO: World Health Organization
ESMO: European Society for Medical Oncology
NCCN: National Comprehensive Cancer Network
CNS: Central Nervous System
TNBC: Triple-Negative Breast Cancer
IBC: Inflammatory Breast Cancer
NF-κB: Nuclear Factor-Kappa B
EGFR: Epidermal Growth Factor Receptor
HIF1: Hypoxia-Inducible Factor-1
DUSP9: Dual Specificity Phosphatase
GSK3β: Glycogen Synthase Kinase 3 Beta
PD-1: Programmed Death 1
KIF11: Kinesin Family Member 11

## References

[1] Bray F Ferlay J Soerjomataram I Siegel RL Torre LA Jemal A Global cancer statistics 2018: GLOBOCAN estimates of incidence and mortality worldwide for 36 cancers in 185 countries CA Cancer J Clin 2018 68 394 424 30207593 10.3322/caac.21492

[2] Ehmsen S Ditzel HJ Signaling pathways essential for triple-negative breast cancer stem-like cells StemCell 2021 39 133 43 10.1002/stem.3301 33211379

[3] Clusan L Ferrière F Flouriot G Pakdel F A basic review on estrogen receptor signaling pathways in breast cancer Int J Mol Sci 2023 6 6834 10.3390/ijms24076834 PMC10095386 37047814

[4] Liang Y Zhang H Song X Yang Q Metastatic heterogeneity of breast cancer: molecular mechanism and potential therapeutic targets Semin Cancer Biol 2020 60 14 27 31421262 10.1016/j.semcancer.2019.08.012

[5] Malik P Chaudhry N Mittal R Mukherjee TK Role of receptor for advanced glycation end products in the complication and progression of various types of cancers Biochim Biophys Acta Gen Subj 2015 1850 1898 904 10.1016/j.bbagen.2015.05.020 26028296

[6] Al-Hajj M Wicha MS Benito-Hernandez A Morrison SJ Clarke M Prospective identification of tumorigenic breast cancer cells Proc Natl Acad Sci USA 2003 100 3983 8 12629218 10.1073/pnas.0530291100 PMC153034

[7] Wysocka J New WHO classification of breast tumours–as published in 2019 Nowotwory J Oncol 2020 70 250 2

[8] Perou CM Sørlie T Eisen MB Van DRM Jeffrey SS Rees CA Molecular portraits of human breast tumours Nature 2000 406 747 52 10963602 10.1038/35021093

[9] Łukasiewicz S Czeczelewski M Forma A Baj J Sitarz R Stanisławek A Breast cancer—epidemiology, risk factors, classification, prognostic markers, and current treatment strategies—an updated review Cancers 2021 13 4287 34503097 10.3390/cancers13174287 PMC8428369

[10] Čipak GA Milković L Dandachi N Stanzer S Pezdirc I Vrančić J Chronic oxidative stress promotes molecular changes associated with epithelial mesenchymal transition, NRF2, and breast cancer stem cell phenotype Antioxidants 2019 8 633 31835715 10.3390/antiox8120633 PMC6943739

[11] Farooqi AA Fayyaz S Hou MF Li KT Tang JY Chang HW Reactive oxygen species and autophagy modulation in non-marine drugs and marine drugs Mar Drugs 2014 12 5408 24 25402829 10.3390/md12115408 PMC4245538

[12] Parker JS Mullins M Cheang MC Leung S Voduc D Vickery T Supervised risk predictor of breast cancer based on intrinsic subtypes J Clin Oncol 2023 41 4192 9 37672882 10.1200/JCO.22.02511

[13] Mittal R Chaudhry N Pathania S Mukherjee TK Mechanistic insight of drug resistance with special focus on iron in estrogen receptor positive breast cancer Curr Pharmaceut Biotechnol 2014 15 1141 57 10.2174/1389201015666141126121240 25429654

[14] Prat A Cheang MC Martín M Parker JS Carrasco E Caballero R Prognostic significance of progesterone receptor–positive tumor cells within immunohistochemically defined luminal A breast cancer J Clin Oncol 2013 31 203 23233704 10.1200/JCO.2012.43.4134 PMC3532392

[15] Balkrishna A Kumar S Hazari P Sahu R Arya V Computational analysis depicting potency of phytochemicals to target MAPK signalling pathway in breast cancer J Nat Remedies 2023 23 923 36

[16] Garrido-Castro AC Lin NU Polyak K Insights into molecular classifications of triple-negative breast cancer: improving patient selection for treatment Cancer Discov 2019 9 176 98 30679171 10.1158/2159-8290.CD-18-1177 PMC6387871

[17] Prat A Pineda E Adamo B Galván P Fernández A Gaba L Clinical implications of the intrinsic molecular subtypes of breast cancer T Breast 2015 24 Suppl 2 S26 35 10.1016/j.breast.2015.07.008 26253814

[18] Orrantia-Borunda E Anchondo-Nuñez P Acuña-Aguilar LE Gómez-Valles FC Ramírez-Valdespino CA Subtypes of breast cancer Mayrovitz HN Breast Cancer Brisbane Exon Publications 2022 31 42 36122153

[19] Higgins MJ Stearns V Understanding resistance to tamoxifen in hormone receptor–positive breast cancer Clin Chem 2009 55 1453 5 19541862 10.1373/clinchem.2009.125377

[20] Loibl S Gianni L HER2-positive breast cancer Lancet 2017 389 2415 29 27939064 10.1016/S0140-6736(16)32417-5

[21] Zhou GQ Lv JW Tang LL Mao YP Guo R Ma J Evaluation of the national comprehensive cancer network and European society for medical oncology nasopharyngeal carcinoma surveillance guidelines Front Oncol 2020 10 119 32117766 10.3389/fonc.2020.00119 PMC7034102

[22] Paluch-Shimon S Cherny NI de Vries EGE Dafni U Piccart MJ Latino NJ Application of the ESMO-Magnitude of Clinical Benefit Scale (V.1.1) to the field of early breast cancer therapies ESMO Open 2020 5 e000743 32893189 10.1136/esmoopen-2020-000743 PMC7476474

[23] Matro JM Li T Cristofanilli M Hughes ME Ottesen RA Weeks JC Inflammatory breast cancer management in the national comprehensive cancer network: the disease, recurrence pattern, and outcome Clin Beast Cancer 2015 15 1 7 10.1016/j.clbc.2014.05.005 PMC4422394 25034439

[24] Inic Z Zegarac M Inic M Markovic I Kozomara Z Djurisic I Difference between luminal A and luminal B subtypes according to Ki-67, tumor size, and progesterone receptor negativity providing prognostic information Clin Med Insights Oncol 2014 8 107 11 25249766 10.4137/CMO.S18006 PMC4167319

[25] Lafcõ O Celepli P Öztekin PS Koşar PN DCE-MRI Radiomics analysis in differentiating luminal a and luminal B breast cancer molecular subtypes Acad Radiol 2023 30 22 9 35595629 10.1016/j.acra.2022.04.004

[26] Aznab M Shojae S Sorkheh AG Rezaei M The survival of patients with triple negative breast cancer undergoing chemotherapy along with lifestyle change interventions: survival of TNBC patients Arch Breast Cancer 2023 10 66 73

[27] Balkrishna A Mittal R Arya V Unveiling novel therapeutic drug targets and prognostic markers of triple negative breast cancer Curr Cancer Drug Targets 2021 21 907 18 34503412 10.2174/1568009621666210908113010

[28] Park S Koo JS Kim MS Park HS Lee JS Lee JS Characteristics and outcomes according to molecular subtypes of breast cancer as classified by a panel of four biomarkers using immunohistochemistry Breast 2012 21 50 7 21865043 10.1016/j.breast.2011.07.008

[29] Chaytow H Carroll E Gordon D Huang YT Van Der Hoorn D Smith HL Targeting phosphoglycerate kinase 1 with terazosin improves motor neuron phenotypes in multiple models of amyotrophic lateral sclerosis EBioMedicine 2022 83 104202 35963713 10.1016/j.ebiom.2022.104202 PMC9482929

[30] Yin L Duan JJ Bian XW Yu SC Triple-negative breast cancer molecular subtyping and treatment progress Breast Cancer Res 2020 22 1 3 10.1186/s13058-020-01296-5 PMC7285581 32517735

[31] Wolff AC Hammond ME Hicks DG Dowsett M McShane LM Allison KH Recommendations for human epidermal growth factor receptor 2 testing in breast cancer: American Society of Clinical Oncology/College of American Pathologists clinical practice guideline update J Clin Oncol 2013 31 3997 4013 24101045 10.1200/JCO.2013.50.9984

[32] Perou CM Sørlie T Eisen MB Van DRM Jeffrey SS Rees CA Molecular portraits of human breast tumours Nature 2000 406 747 52 10963602 10.1038/35021093

[33] Prat A Pineda E Adamo B Galván P Fernández A Gaba L Clinical implications of the intrinsic molecular subtypes of breast cancer Breast 2015 24 10.1016/j.breast.2015.07.008 26253814

[34] Morris GJ Naidu S Topham AK Guiles F Xu Y McCue P Differences in breast carcinoma characteristics in newly diagnosed African-American and Caucasian patients: a single-institution compilation compared with the National Cancer Institute's Surveillance, Epidemiology, and End Results database Cancer 2007 110 876 84 17620276 10.1002/cncr.22836

[35] Dent R Trudeau M Pritchard KI Hanna WM Kahn HK Sawka CA Triple-negative breast cancer: clinical features and patterns of recurrence Clin Cancer Res 2007 13 4429 34 17671126 10.1158/1078-0432.CCR-06-3045

[36] Lin NU Claus E Sohl J Razzak AR Arnaout A Winer EP Sites of distant recurrence and clinical outcomes in patients with metastatic triple-negative breast cancer: high incidence of central nervous system metastases Cancer 2008 113 2638 45 18833576 10.1002/cncr.23930 PMC2835546

[37] Zhang L Fang C Xu X Li A Cai Q Long X Androgen receptor, EGFR, and BRCA1 as biomarkers in triple-negative breast cancer: a meta-analysis BioMed Res Int 2015 2015 357485 25695063 10.1155/2015/357485 PMC4324735

[38] Gluz O Liedtke C Gottschalk N Pusztai L Nitz U Harbeck N Triple-negative breast cancer–current status and future directions Ann Oncol 2009 20 1913 27 19901010 10.1093/annonc/mdp492

[39] Chaudhary LN Wilkinson KH Kong A Triple-negative breast cancer: who should receive neoadjuvant chemotherapy? Surg Oncol Clin N Am 2018 27 141 53 29132557 10.1016/j.soc.2017.08.004

[40] Collignon J Lousberg L Schroeder H Jerusalem G Triple-negative breast cancer: treatment challenges and solutions Breast Cancer (Dove Med Press) 2016 8 93 107 27284266 10.2147/BCTT.S69488 PMC4881925

[41] Wang W Nag SA Zhang R Targeting the NFκB signaling pathways for breast cancer prevention and therapy Curr Med Chem 2015 22 264 89 25386819 10.2174/0929867321666141106124315 PMC6690202

[42] Romieu-Mourez R Kim DW Shin SM Demicco EG Landesman-Bollag E Seldin DC Mouse mammary tumor virus c-rel transgenic mice develop mammary tumors Mol Cell Biol 2003 23 5738 54 12897145 10.1128/MCB.23.16.5738-5754.2003 PMC166341

[43] Liu M Sakamaki T Casimiro MC Willmarth NE Quong AA Ju X The canonical NFkappaB pathway governs mammary tumorigenesis in transgenic mice and tumor stem cell expansion Cancer Res 2010 70 10464 73 21159656 10.1158/0008-5472.CAN-10-0732 PMC3010731

[44] Taipale J Beachy PA The Hedgehog and Wnt signaling pathways in cancer Nature 2001 411 349 54 11357142 10.1038/35077219

[45] Hammerschmidt M Brook A McMahon AP The world according to hedgehog Trends Genet 1997 13 14 21 9009843 10.1016/s0168-9525(96)10051-2

[46] Malanga D Belmonte S Colelli F Scarfò M De MC Oliveira DM AKT1E17K is oncogenic in mouse lung and cooperates with Chemical Carcinogens in inducing Lung Cancer PLoS One 2016 11 e0147334 26859676 10.1371/journal.pone.0147334 PMC4747507

[47] Paplomata E O'Regan R The PI3K/AKT/mTOR pathway in breast cancer: targets, trials and biomarkers Ther Adv Med Oncol 2014 6 154 66 25057302 10.1177/1758834014530023 PMC4107712

[48] Cantley LC The phosphoinositide 3-kinase pathway Science 2002 296 1655 7 12040186 10.1126/science.296.5573.1655

[49] Klarenbeek S van Miltenburg MH Jonkers J Genetically engineered mouse models of PI3K signaling in breast cancer Mol Oncol 2013 7 146 64 23478237 10.1016/j.molonc.2013.02.003 PMC5528412

[50] Li Q Li Z Luo T Shi H Targeting the PI3K/AKT/mTOR and RAF/MEK/ERK pathways for cancer therapy Mol Biomed 2022 3 47 36539659 10.1186/s43556-022-00110-2 PMC9768098

[51] Gasparri ML Besharat ZM Farooqi AA Khalid S Taghavi K Besharat RA MiRNAs and their interplay with PI3K/AKT/mTOR pathway in ovarian cancer cells: a potential role in platinum resistance J Cancer Res Clin Oncol 2018 144 2313 8 30109500 10.1007/s00432-018-2737-y PMC11813288

[52] Balkrishna A Mittal R Arya V Unveiling role of MicroRNAs as treatment strategy and prognostic markers in triple negative breast cancer Curr Pharmaceut Biotechnol 2020 21 1569 75 10.2174/1389201021666200627201535 32593278

[53] Engelman JA Luo J Cantley LC The evolution of phosphatidylinositol 3-kinases as regulators of growth and metabolism Nat Rev Genet 2006 7 606 19 16847462 10.1038/nrg1879

[54] Hu X Li J Fu M Zhao X Wang W The JAK/STAT signaling pathway: from bench to clinic Signal Transduct Targeted Ther 2021 6 402 10.1038/s41392-021-00791-1 PMC8617206 34824210

[55] Owen KL Brockwell NK Parker BS JAK-STAT signaling: a double-edged sword of immune regulation and cancer progression Cancers 2019 11 12 2002 10.3390/cancers11122002 PMC6966445 31842362

[56] Dwyer AR Truong TH Ostrander JH Lange CA 90 years of progesterone: steroid receptors as MAPK signaling sensors in breast cancer: let the fates decide J Mol Endocrinol 2020 65 35 48 32209723 10.1530/JME-19-0274 PMC7329584

[57] Dwyer AR Truong TH Ostrander JH Lange CA 90 years of progesterone: steroid receptors as MAPK signaling sensors in breast cancer: let the fates decide J Mol Endocrinol 2020 65 T35 48 32209723 10.1530/JME-19-0274 PMC7329584

[58] MacNeil AJ Jiao SC McEachern LA Yang YJ Dennis A Yang YJ MAPK kinase 3 is a tumor suppressor with reduced copy number in breast cancer Cancer Res 2014 74 162 72 24233520 10.1158/0008-5472.CAN-13-1310

[59] Issinger OG Guerra B Phytochemicals in cancer and their effect on the PI3K/AKTmediated cellular signalling Biomed Pharmacother 2021 139 111650 33945911 10.1016/j.biopha.2021.111650

[60] Aittomäki S Pesu M Therapeutic targeting of the Jak/STAT pathway Basic Clin Pharmacol Toxicol 2014 114 18 23 24164900 10.1111/bcpt.12164

[61] Yin P Wang W Zhang Z Bai Y Gao J Zhao C Wnt signaling in human and mouse breast cancer: focusing on Wnt ligands, receptors and antagonists Cancer Sci 2018 109 3368 75 30137666 10.1111/cas.13771 PMC6215866

[62] Braune EB Seshire A Lendahl U Notch and Wnt dysregulation and its relevance for breast cancer and tumor initiation Biomedicines 2018 6 101 30388742 10.3390/biomedicines6040101 PMC6315509

[63] Pandey P Khan F Seifeldin SA Alshaghdali K Siddiqui S Abdelwadoud ME Targeting Wnt/β-catenin pathway by flavonoids: implication for cancer therapeutics Nutrients 2023 15 9 2088 10.3390/nu15092088 PMC10181252 37432240

[64] Xin P Xu X Deng C Liu S Wang Y Zhou X The role of JAK/STAT signaling pathway and its inhibitors in diseases Int Immunopharm 2020 80 106210 10.1016/j.intimp.2020.106210 31972425

[65] Evans MK Brown MC Geradts J Bao X Robinson TJ Jolly MK XIAP regulation by MNK links MAPK and NFκB signaling to determine an aggressive breast cancer phenotype Cancer Res 2018 78 1726 38 29351901 10.1158/0008-5472.CAN-17-1667 PMC7724565

[66] Xu M Wang S Wang Y Wu H Frank JA Zhang Z Role of p38γ MAPK in regulation of EMT and cancer stem cells Biochim Biophys Acta Mol 2018 1864 3605 17 10.1016/j.bbadis.2018.08.024 PMC6428452 30251680

[67] Jang GB Kim JY Cho SD Park KS Jung JY Lee HY Blockade of Wnt/βcatenin signaling suppresses breast cancer metastasis by inhibiting CSC-like phenotype Sci Rep 2015 5 12465 26202299 10.1038/srep12465 PMC5378883

[68] Balkrishna A Mittal R Arya V Unveiling role of MicroRNAs in metastasizing triple negative breast cancer: from therapeutics to delivery Curr Drug Targets 2023 24 509 20 36892021 10.2174/1389450124666230308154551

[69] Takebe N Miele L Harris PJ Jeong W Bando H Kahn M Targeting Notch, Hedgehog, and Wnt pathways in cancer stem cells: clinical update Nat Rev Clin Oncol 2015 12 445 64 25850553 10.1038/nrclinonc.2015.61 PMC4520755

[70] Yan Y Liu F Han L Zhao L Chen J Olopade OI HIF-2α promotes conversion to a stem cell phenotype and induces chemoresistance in breast cancer cells by activating Wnt and Notch pathways J Exp Clin Cancer Res 2018 37 256 30340507 10.1186/s13046-018-0925-x PMC6194720

[71] Vijay GV Zhao N Den HP Toneff MJ Joseph R Pietila M GSK3β regulates epithelial-mesenchymal transition and cancer stem cell properties in triple-negative breast cancer Breast Cancer Res 2019 21 37 30845991 10.1186/s13058-019-1125-0 PMC6407242

[72] Simeone P Trerotola M Franck J Cardon T Marchisio M Fournier I The multiverse nature of epithelial to mesenchymal transition Semin Cancer Biol 2019 58 1 10 30453041 10.1016/j.semcancer.2018.11.004

[73] Chaurasia M Singh R Sur S Flora SJS A review of FDA approved drugs and their formulations for the treatment of breast cancer Front Pharmacol 2023 14 1184472 37576816 10.3389/fphar.2023.1184472 PMC10416257

[74] Sarika PR Nirmala RJ Curcumin loaded gum Arabic aldehyde-gelatin nanogels for breast cancer therapy Mater Sci Eng C Mater Biol Appl 2016 65 331 7 27157759 10.1016/j.msec.2016.04.044

[75] Attama AA Nnamani PO Onokala OB Ugwu AA Onugwu AL Nanogels as target drug delivery systems in cancer therapy: a review of the last decade Front Pharmacol 2022 13 874510 36160424 10.3389/fphar.2022.874510 PMC9493206

[76] McGrowder DA Miller FG Nwokocha CR Anderson MS Wilson-Clarke C Vaz K Medicinal herbs used in traditional management of breast cancer: mechanisms of action Medicines 2020 7 47 32823812 10.3390/medicines7080047 PMC7460502

[77] Baraya YS Wong KK Yaacob NS The Immunomodulatory potential of selected bioactive plant-based compounds in breast cancer: a review Anti Cancer Agents Med Chem 2017 17 770 83 10.2174/1871520616666160817111242 27539316

[78] Křížová L Dadáková K Kašparovská J Kašparovský T Isoflavones Molecules 2019 24 1076 30893792 10.3390/molecules24061076 PMC6470817

[79] Tanriover G Dilmac S Aytac G Farooqi AA Sindel M Effects of melatonin and doxorubicin on primary tumor and metastasis in breast cancer model Anti Cancer Agents Med Chem 2022 22 1970 83 10.2174/1871520621666211213094258 34961467

[80] Dietz BM Hajirahimkhan A Dunlap TL Bolton JL Botanicals and their bioactive phytochemicals for women's health Pharmacol Rev 2016 68 1026 73 27677719 10.1124/pr.115.010843 PMC5050441

[81] Bak MJ Gupta Das Wahler J Suh N Role of dietary bioactive natural products in estrogen receptor-positive breast cancer Semin Cancer Biol 2016 40 170 91 27016037 10.1016/j.semcancer.2016.03.001 PMC5033666

[82] Balkrishna A Mittal R Arya V Potential role of miRNA in metastatic cascade of triple-negative breast cancer Curr Cancer Drug Targets 2021 21 153 62 33155912 10.2174/1568009620999201103201626

[83] Farooqi AA Qureshi MZ Khalid S Attar R Martinelli C Sabitaliyevich UY Regulation of cell signaling pathways by berberine in different cancers: searching for missing pieces of an incomplete jig-saw puzzle for an effective cancer therapy Cancers 2019 11 478 30987378 10.3390/cancers11040478 PMC6521278

